# SynNER: syntax-infused named entity recognition in the biomedical domain

**DOI:** 10.1093/jamiaopen/ooaf149

**Published:** 2026-02-21

**Authors:** Muhammad Imran, Olga Zamaraeva, Carlos Gómez-Rodríguez

**Affiliations:** Universidade da Coruña, CITIC, Departamento de Ciencias de la Computación y Tecnologías de la Información, Campus de Elviña s/n, A Coruña 15071, Spain; Universidade da Coruña, CITIC, Departamento de Ciencias de la Computación y Tecnologías de la Información, Campus de Elviña s/n, A Coruña 15071, Spain; Universidade da Coruña, CITIC, Departamento de Ciencias de la Computación y Tecnologías de la Información, Campus de Elviña s/n, A Coruña 15071, Spain

**Keywords:** named entity recognition, dependency parsing, sequence labelling

## Abstract

**Objective:**

This study evaluates the usefulness of explicit syntactic knowledge, integrated via a neural mechanism, in improving the accuracy of named entity recognition in the domain of biomedical text processing.

**Materials and Methods:**

Syntactic structure of a text can be helpful to determine whether a certain part of the text is an entity or not. Parsing is an essential technique in natural language processing (NLP) that can be utilized to determine the syntactic structure of sentences in human languages. We propose to infuse syntactic knowledge through the attention mechanism using dependency parsing and sequence labelling parsing, as well as the multi-task learning paradigm. Experiments were conducted on five datasets: MTSamples, VAERS, NCBI-disease, BC2GM, and JNLPBA.

**Results:**

We demonstrate improvements in the F1 score over the current state of the art on 3 out of 5 datasets (MTSamples, VAERS, and NCBI).

**Discussion:**

We reduce the number of mismatches with gold labels in particular in the n-dash and parentheses tokens and in compound and adjective modifier dependencies.

**Conclusion:**

Syntactic features improve NER accuracy in attention-based neural systems, and parsing as sequence labelling brings additional benefits.

## Background and significance

Named entity recognition (NER) is a fundamental task in natural language processing (NLP). It is concerned with detecting portions of text which refer to persons, institutions, locations, substances, and generally entities characterized by having a name. Through the history of NLP, NER has been attempted with a variety of methods, starting from rule-based pipelines (see Grishman et al. 1996^1^ for the earliest mention of the term) and recently, with large language models (LLMs) such as GPT-4 (see Li et al.^2^ for an overview of deep learning approaches in NER). While highly capable, generative LLMs often do not achieve the same level of precision as specialized encoder-based architectures for tasks requiring high precision, like NER, which is concerned with detecting precise spans of entities in the text. For that reason, despite the overwhelming interest in solving NER with LLMs as part of the generalized AI aspirations, work continues on developing specialized NLP pipelines for NER. For now, the best solutions include fine-tuning, feature engineering, and generally multi-component pipelines.

This is particularly true in biomedical NER, where distributions of entities can differ noticeably between the training data for language models and the downstream task datasets, leading to low scores in zero-shot and even multi-shot prompting of LLMs including GPT-4. Fine-tuning remains necessary, but even with fine-tuning, many datasets remain challenging for the current state of the art. This motivates additional components on top of fine-tuning.

In NLP, **dependency parsing** is one of the robust ways to enrich text representation, available now for many languages and independent from the text domain. For NER in particular, using parsing is underexplored, although the nature of syntactic dependencies should, in principle, be beneficial for determining correct entity spans better. Furthermore, parsing can be thought of as sequence labelling,^3^^,^^4^ making it readily applicable in NER. This is what the experiments presented in this paper demonstrate: we integrate the syntactic structures (obtained by dependency parsing) into the attention mechanism using relational graph convolutional networks and sequence labelling encodings, which allows weighing the contribution of syntactic information for each token. This novel approach allows us to beat the state of the art on three out of five datasets (MTSamples, VAERS, NCBI), showing in particular the efficacy of sequence labelling parsing.

## State of the art

Recent work on named entity recognition (NER) in the biomedical domain has been focusing on using large language models (LLM),^5^ despite BioBERT+BiLSTM still showing some of the best results.^6^^,^^7^ Trying to solve as many different problems as possible with generative AI showing its general capacities is a general trend. In the case of NER, the pretrained LLMs are either fine-tuned (trained additionally for the task) or prompted as they are. Fine-tuning is expensive and does not help as much to show AI’s generalizability, but tends to yield better results. As one example, the proposed Category Semantic Enhanced framework for Named Entity Recognition (CSE-NER) uses contrastive learning to inject category semantics into the fine-tuning process, mitigating domain shift.^8^ Another study demonstrated that ensembling fine-tuned LLMs with traditional deep learning models yields superior performance for extracting complex entities like adverse events.^9^ To address computational demands, one study created compact biomedical transformers using knowledge distillation, achieving near-par performance with their larger counterparts.^10^ Prompting in NER is usually defined by the number of examples supplied to the LLM through the prompt: zero-shot (no examples provided) to N-shot where N typically ranges from 1 to 5 but can reach 50 and more.^11^ In addition to using stand-alone LLMs (fine-tuned or not), NER uses pipelines of tools. This continues to be necessary, due to distributional differences between the training data and the downstream task, and since generative LLMs are currently not very good at tasks requiring precision (such as locating an entity’s precise span). Pipeline components include multi-task learning (separate fine-tune stages), feature engineering (adding POS-tags, word embedding) in combination with specialized language models,^6^^,^^7^^,^^12^ data augmentation,^13^ and information retrieval (additional examples of contexts in which entities can be found).^14^ Information retrieval-based pipelines tend to yield higher accuracies than methods with no retrieval, however they depend on the availability of additional information. Also using information retrieval, a practical tool (GPDminer) leverages a BERT-based engine for end-to-end NER and relation extraction in biomedical literature.^15^

Using syntactic knowledge and the related parsing methodologies is underexplored in NER, and shows substantial promise, since constituencies and dependencies are direct cues for correct entity boundaries. Some early examples of using parsing in NER incorporated syntactic features in the NER pipelines of the day, showing improvements.^16–18^ In the neural era, linguistic features obtained with shallow parsing (chunking) were shown to improve accuracy for entity spans, however at the time, they were only able to show this effect for neural NER models without the attention mechanism.^19^ More recently, syntactic parses were integrated into the attention mechanism by reframing the NER task as a graph node classification problem, showing improvements on eight benchmark datasets.^20^ This work is an example of thinking of syntactic information as motivating a more structural look at the problem of NER itself; see also Tian et al.,^21^ who were one of the first to incorporate syntactic information into a NER pipeline not by simply concatenating it to data representations but in the form of key-value memory networks. In another study,^22^ authors report notably high performance for their AIMFF model. Their description of the evaluation suggests that the reported F1 scores may be based on token-level alignment rather than entity-based metrics, as they refer to “words” within entities and do not specify whether strict or relaxed entity-level criteria were applied. Since no public code is available for verification, we do not include their results as we do not know if they are comparable to our entity-based metrics or rather to our token-level evaluation.

More recent work showed that parsing can be thought of as sequence labelling,^3^^,^^4^ and so can NER, which makes the connection between the two more readily natural.^23^ Our study combines modern attention-based architectures with the view on parsing as sequence labelling, while also reproducing some baseline results with language models fine-tuning and with traditional dependency parsing.

## Materials and methods

### Datasets

In this study, we use five biomedical benchmark datasets described in Chen et al.^24^ (Table 1).

**Table 1.** Datasets overview

Dataset	Train	Dev	Test	Entity Type
MTSamples	602	201	201	Medical Transcriptions
VAERS	603	126	286	Nervous System Disorder
NCBI	4373	1,457	1,457	Disease
BC2GM	12,079	4,026	4,026	Gene/Protein
JNLPBA	14,884	4,961	4,961	Gene/Protein, Cell

Table 2 summarizes the state of the art on five benchmark datasets. Our study trains models without using any external data beyond the datasets’ training sets. Thus, following standard evaluation practices, our results are directly comparable to those from fine-tuning, but should not be compared against results from ensemble methods or systems that use additional data sources, such as information retrieval pipelines. The F1 scores are entity-based strict match, so the entity is counted as correctly recognized only if the entire span and the type of the entity was correctly identified (cf. token-based evaluation where every token belonging to a name of an entity counts towards the score).^1^

**Table 2.** State of the art for different NER methods on biomedical datasets used in our experiments; strict entity-based metric

Dataset	Method	F1-score	Reported by
MTSamples	GPT-4 prompting	0.593	Hu et al. 2024^6^
	BioClinicalBert fine tuning	0.785	Hu et al. 2024^6^
VAERS	GPT-4 prompting	0.542	Hu et al. 2024^6^
	BioClinicalBert fine tuning	0.668	Hu et al. 2024^6^
	GPT-3.5 fine tuning, **ensemble**	0.781	Li et al. 2025^9^
NCBI	LLM 50-shot prompting	0.726	Mu et al. 2024^11^
	LLM fine tuning with multi-task learning	0.899	Dai et al. 2024^8^
	BERT+BiLSTM+CRF with feature engineering	0.9166	Alamro et al. 2024^7^
	LLM + **information retrieval**	0.9176	Li et al. 2024^14^
BC2GM	Syntax features; NER as node classification	0.8515	Zheng et al. 2022^20^
	**Information retrieval**	0.8719	Park et al. 2024^15^
	Instruction tuning, GPT-4	0.8762	Rohanian et al. 2024^10^
	BioBERT+BiLSTM with embedding	0.8912	Alamro et al. 2024^7^
JNLPBA	Syntax features; NER as node classification	0.7816	Zheng et al. 2022^20^
	BioBERT+BiLSTM with embedding	0.7939	Alamro et al. 2024^7^
	Instruction tuning, GPT-4	0.8230	Rohanian et al. 2024^10^
	BiLSTM+CRF with pretrained word embedding	0.8432	Dash et al. 2022^12^

### Methods

To incorporate syntactic knowledge into NER, we propose a hybrid model that integrates dependency parsing information into a transformer-based architecture through a graph attention mechanism. Specifically, we leverage dependency tree (dependency heads and relations following the Universal Dependencies annotation scheme;^26^  Figure 2) to construct a graph structure over tokens and apply a Relational Graph Attention Network (RGAT^27^) to model syntax-aware token interactions. We used the spaCy parser (general-purpose) to obtain dependency trees and encoded them under a sequence labelling setup^28^ using Relative and Absolute Encoding linearization techniques.^4^ While this parser is not optimized for biomedical text, its output served as an effective syntactic feature in our model; employing a biomedically-trained parser presents a promising avenue for future performance gains.

### Syntactic parsing

**Figure 1.**
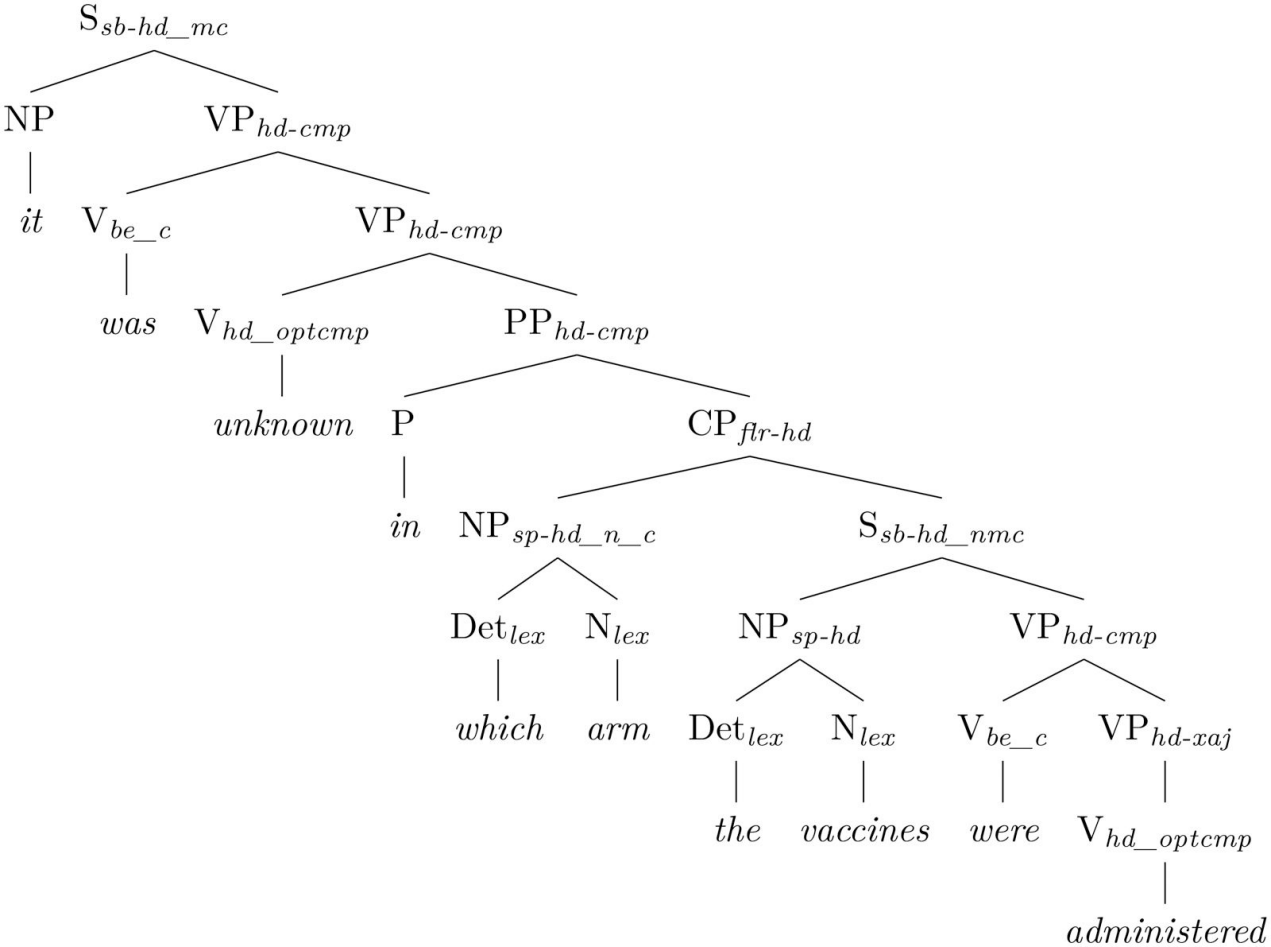
A constituency parse.

Syntactic parsing is mapping sentence strings to their underlying structural representations. In linguistics, the study of sentence structure is called syntax, and parsing is the essential part of studying and using syntax in natural language processing (NLP). Parsing can be done by explicitly modeling grammar rules, which allows for internal consistency and interpretability, and by training statistical and neural systems, which allows for complete coverage of the data and overall robustness. For downstream tasks, such as NER, robustness is key. The two primary approaches to parsing are constituency and dependency parsing. **Constituency parsing** is a way of analyzing a sentence by organizing its parts into a tree structure that shows how they group together to form meaningful components (see Figure 1). For example, words like *the vaccines* form a noun phrase—a group of words that together act like a noun. Similarly, *administered in the left arm* is a verb phrase, acting like a verb. The parser builds a hierarchy where each group, or constituent, reflects how the sentence is structured grammatically. Figure 1 shows the English Resource Grammar^29^^,^^30^ constituency visualization of the analysis of the sentence *It was unknown in which arm the vaccines were administered* (VAERS 2025). The constituency parse shows that the sentence contains such noun phrases as *the vaccines* and *which arm*, that *the vaccines* combine with the verb phrase *were administered* as its subject, and so on.

**Figure 2.**
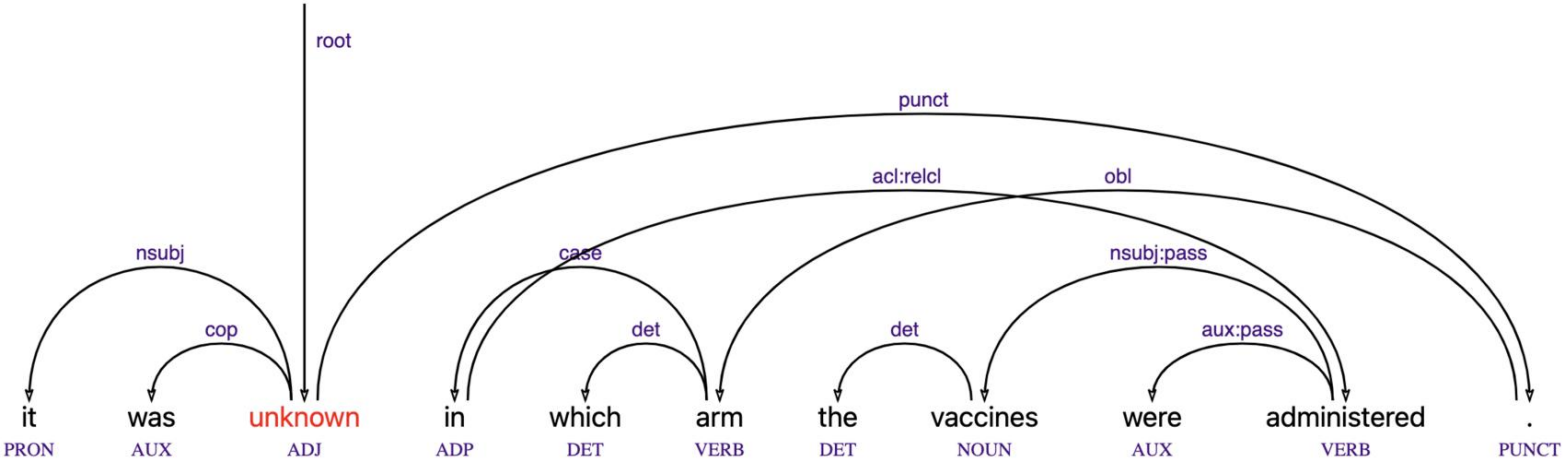
A dependency parse.

Constituency parsing is complex and slow. While it remains important in some areas, in many downstream tasks **dependency parsing** has long been considered an efficient alternative. Dependency parsing maps the sentence string to an underlying graph of relations between parts of the sentence. Figure 2 shows a dependency analysis for the same sentence, obtained by the Salsa system.^2^^,^^31^ While a dependency parse does not directly represent constituents, some constituency information can be reconstructed from it and used for boosting precision in recognizing named entity spans. In many cases, dependencies are even more important for detecting such spans than constituency, for example long distance dependencies (where some parts of a constituent can be separated from it). In the example sentence, *in which arm* is modifying the verb *administered* and, in a canonical sentence not containing an embedded question, it would directly follow the verb (eg, *The vaccines were administered in the left arm*). However, in the example, it is separated from the verb by the noun phrase *the vaccines*.

**Parsing as sequence labelling** ^3^ ^,^ ^4^ is a modern approach to constituency and dependency parsing which reframes parsing as a more generic task, connecting it to the array of NLP tasks solved successfully with neural architectures. The essence of parsing as sequence labelling is linearizing sentence structure such that it is presented as a string of words where each word is labeled (in a one-to-one correspondence) with a piece of syntactic information. When combined, this sequence of labels can be used to reconstruct each word’s grammatical role and the sentence’s overall structure (ie, the constituent or dependency tree).

### Model architecture

**Figure 3.**
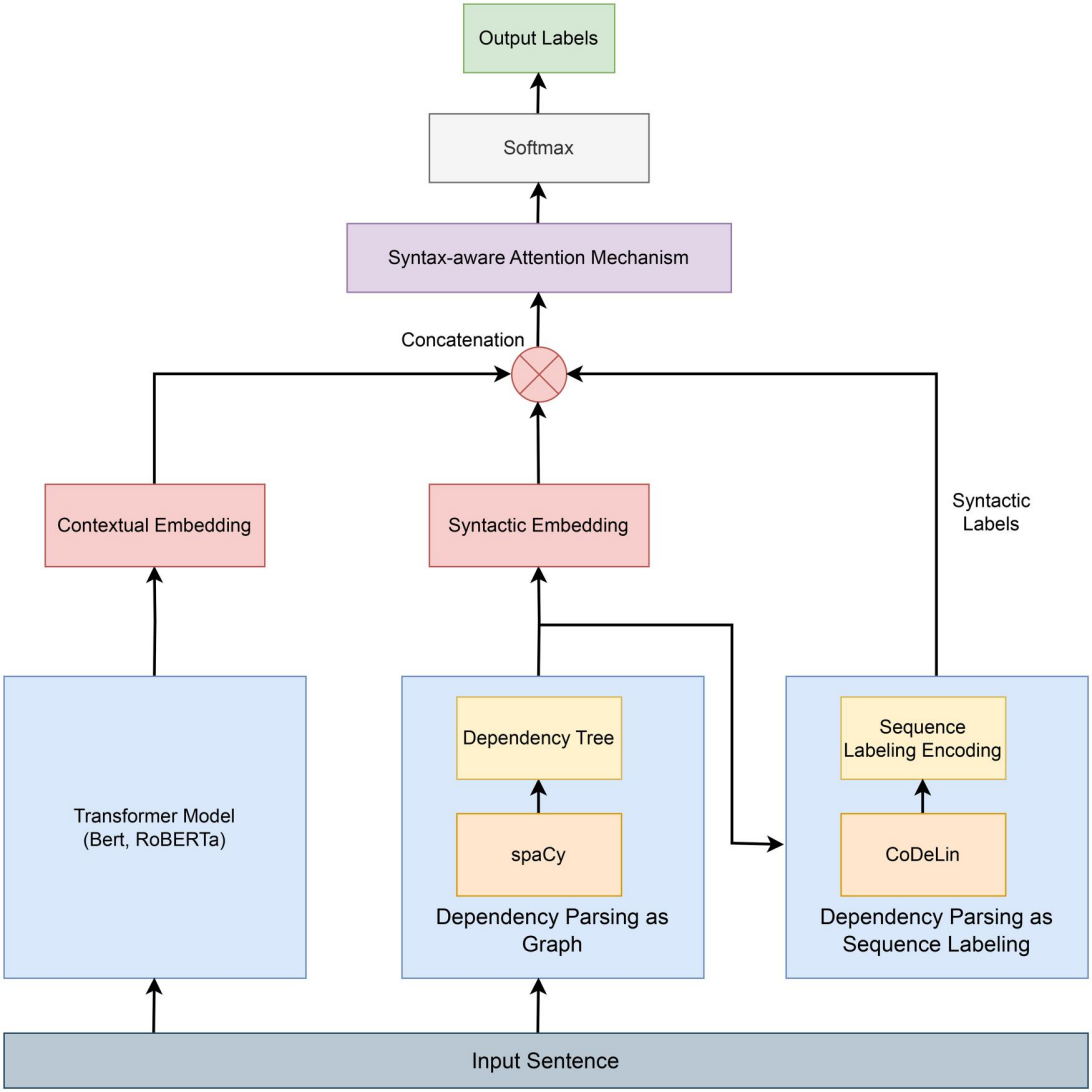
Overall system architecture.

The model architecture (Figure 3) is built around two complementary pathways: one that provides contextual embeddings derived from a pretrained transformer backbone, and another that uses a dependency parser to obtain syntactic relationships and explicitly encodes them, either directly as a dependency graph or through sequence labelling. These syntactic relationships are then fused (through a syntax-aware attention mechanism) to produce syntax-aware token-level embeddings for NER.

In the first pathway, the input sentence is encoded by a pretrained transformer model (BERT, RoBERTa), whose final hidden states provide rich contextual representations for each token. Simultaneously, in the second pathway, the system first obtains syntactic relationships for the sentence through dependency parsing (spaCy). These syntactic relationships are then incorporated into the model via one of two alternative approaches. (1) In the first approach, the system constructs a dependency graph directly for the dependency parse tree, where tokens are connected according to their grammatical relationships. A multi-headed RGAT^27^ then propagates information along these edges, allowing the model to capture higher-order syntactic patterns. The output of this graph network yields structured embeddings that emphasize how tokens function within the overall sentence structure. (2) In the second approach, the dependency parse trees are converted into a sequence of syntactic labels using a sequence labelling framework CoDeLin.^32^ For each token, the syntactic label (head position, dependency relation) is converted into an embedding vector (eg, relation label embedding). These embedding reflect the positional relationship (using relative encoding or absolute encoding) between each token and its syntactic head, ensuring that local structural cues are available.

The system combines these complementary pathways through a lightweight syntax-aware attention mechanism that learns, for each token, the appropriate balance between its contextual embedding and its syntactic embedding. The two representations (contextual and syntactic) are merged via a learned weighting mechanism, enabling the model to emphasize contextual cues when they are most informative and to fall back on syntactic signals when they better capture entity boundaries.

Finally, the fused token representations are fed into a standard classification layer that assigns a probability distribution over possible entity labels for each token. Thus, our system **SynNER** combines both context and syntax in a simple, trainable model to provide more accurate named entity predictions.

### Syntax-aware attention mechanism

The attention mechanism is a learned fusion mechanism designed to integrate semantic features from a pretrained transformer (eg, BERT, RoBERTa) with syntactic features derived from dependency parsing tree, modelled through RGAT. This mechanism allows the model to dynamically weigh the contribution of semantic and syntactic information for each token during the Named Entity Recognition (NER) task.

To effectively integrate semantic and syntactic information, the model employs a *token-level attention mechanism* that dynamically fuses contextual embedding from the transformer model with structure-aware embeddings derived from RGAT. For each token, the semantic vector from the transformer output $h_{i}\in R^{H}$ and the projected syntactic vector from the GAT output $g_{i}\in R^{H}$ are concatenated into a combined feature vector:


$$z_{i}=[h_{i};g_{i}]\in R^{2H}$$

This concatenated vector is passed through a lightweight feedforward attention network consisting of a linear transformation followed by a non-linear activation and a final scoring layer:


$$a_{i}=\text{Softmax}\left(W_{2}\cdot \tanh(W_{1}z_{i}+b_{1})+b_{2}\right)\in R^{2}$$

where $W_{1}\in R^{H\times 2H}$, $W_{2}\in R^{2\times H}$, and $a_{i}=[\alpha_{i}^{\left(h\right)},\alpha_{i}^{\left(g\right)}]$ denotes the attention weights corresponding to the transformer-based (semantic) and GAT-based (syntactic) features, respectively. These attention weights are normalized using the softmax function such that $\alpha_{i}^{\left(h\right)}+\alpha_{i}^{\left(g\right)}=1$. The final fused representation for each token is computed as a weighted combination of the semantic and syntactic features:


$$f_{i}=\alpha_{i}^{\left(h\right)}\cdot h_{i}+\alpha_{i}^{\left(g\right)}\cdot g_{i}$$

The fused representations are then passed through a dropout layer for regularization before being projected into the label space via a linear classifier (classification layer), producing entity type predictions for each token. This attention-based fusion allows the model to dynamically adjust the contribution of semantic and syntactic information for each token, thereby enhancing representation quality and improving performance on the NER task.

### Multi-task learning for NER

An alternative way to incorporate syntax information into NER process is Multi-task learning (MTL). MTL is a powerful framework for improving NER by jointly training it with syntactically related auxiliary tasks such as dependency parsing as sequence labelling. We implement MTL with MaChAmp Toolkit,^33^ where both tasks share a common encoder (a pretrained transformer model) while maintaining separate task-specific decoders. The auxiliary parsing task guides the model to learn syntactic structures, such as head–dependent relations and phrase boundaries, which are especially valuable in the biomedical domain where entities often align with noun phrases or syntactic constituents. By incorporating dependency parsing as sequence labelling as an auxiliary task, the model’s shared representation becomes aware of grammatical roles and structural cues that help identify entity spans.

## Results

Table 3 presents our results. Our baseline is fine-tuned transformer-based models without syntactic features. Comparing to this baseline makes it clear that infusing syntactic information in the form of dependency parsing, particularly when encoded in sequence labelling form, into the attention mechanism brings benefits on all datasets. Furthermore, we beat the relevant SOTA (methods not involving ensemble pipelines; Table 2) on three out of five datasets (MTSamples, VAERS, and NCBI). More detailed results can be found in the Appendix A in supplementary materials.

**Table 3.** Entity-Level F1-Scores (Exact Match)

Dataset	Encoders	Baseline			DP as Graph			DP as Seq. Labeling						Multi-task Learning					
								Rel			Abs			Rel			Abs		
		P	R	F1	P	R	F1	P	R	F1	P	R	F1	P	R	F1	P	R	F1
MTSamples	bert-base-uncased	0.720	0.761	0.740	0.736	0.764	0.750	0.767	0.764	0.765	0.757	0.757	0.757	0.726	0.736	0.731	0.736	0.757	0.747
	distilbert-base-uncased	0.704	0.729	0.716	0.731	0.757	0.744	0.747	0.729	0.738	0.711	0.718	0.715	0.730	0.743	0.736	0.765	0.732	0.748
	roberta-base	0.749	0.746	0.748	0.773	0.754	0.763	0.794	0.799	0.796	0.763	0.792	0.777	0.722	0.739	0.730	0.731	0.736	0.733
	biobert-v1.1	0.708	0.743	0.725	0.779	0.771	0.775	0.773	0.757	0.765	0.804	0.796	**0.800**	0.799	0.785	0.792	0.785	0.771	0.778
	Bio_ClinicalBERT	0.774	0.736	0.755	0.742	0.718	0.730	0.728	0.736	0.732	0.785	0.771	0.778	0.797	0.775	0.786	0.755	0.725	0.740
	BiomedNLP	0.769	0.785	0.777	0.738	0.785	0.761	0.783	0.775	0.779	0.758	0.739	0.749	0.795	0.778	0.786	0.781	0.768	0.774
	SapBERT	0.693	0.754	0.722	0.769	0.750	0.759	0.745	0.771	0.758	0.698	0.750	0.723	0.78	0.799	0.790	0.760	0.771	0.766
		Prior Comparable SOTA (BioClinicalBert fine tuning, Hu et al. 2024^6^) = 0.785																	
VAERS	bert-base-uncased	0.532	0.675	0.595	0.597	0.663	0.629	0.595	0.605	0.600	0.568	0.638	0.601	0.580	0.644	0.610	0.549	0.615	0.58
	distilbert-base-uncased	0.539	0.599	0.568	0.546	0.605	0.574	0.545	0.566	0.555	0.566	0.650	0.605	0.554	0.626	0.588	0.520	0.593	0.555
	roberta-base	0.610	0.708	0.655	0.625	0.716	0.667	0.609	0.708	0.655	0.618	0.710	0.661	0.568	0.669	0.614	0.572	0.648	0.608
	biobert-v1.1	0.588	0.691	0.635	0.612	0.679	0.644	0.576	0.661	0.616	0.608	0.661	0.634	0.647	0.708	0.676	0.608	0.661	0.634
	Bio_ClinicalBERT	0.580	0.665	0.620	0.582	0.681	0.628	0.586	0.689	0.633	0.587	0.687	0.633	0.594	0.648	0.620	0.595	0.652	0.622
	BiomedNLP	0.570	0.652	0.608	0.649	0.708	0.677	0.568	0.695	0.625	0.599	0.698	0.645	0.601	0.667	0.632	0.594	0.667	0.629
	SapBERT	0.606	0.693	0.647	0.653	0.743	**0.695**	0.636	0.722	0.676	0.661	0.730	0.694	0.613	0.698	0.653	0.628	0.691	0.658
		Prior Comparable SOTA (BioClinicalBert fine tuning, Hu et al. 2024^6^) = 0.668																	
NCBI	bert-base-uncased	0.876	0.875	0.876	0.892	0.893	0.893	0.883	0.871	0.876	0.861	0.879	0.896	0.881	0.888	0.885	0.884	0.890	0.887
	distilbert-base-uncased	0.879	0.873	0.876	0.883	0.876	0.879	0.858	0.880	0.875	0.870	0.886	0.872	0.877	0.880	0.879	0.888	0.881	0.884
	roberta-base	0.864	0.894	0.879	0.881	0.909	0.895	0.899	0.899	0.895	0.906	0.896	0.894	0.872	0.882	0.877	0.868	0.877	0.872
	biobert-v1.1	0.893	0.897	0.895	0.894	0.903	0.898	0.894	0.899	0.888	0.900	0.898	0.890	0.891	0.907	0.899	0.894	0.899	0.896
	Bio_ClinicalBERT	0.903	0.881	0.892	0.891	0.892	0.892	0.890	0.901	0.882	0.902	0.876	0.872	0.884	0.883	0.883	0.886	0.875	0.880
	BiomedNLP	0.888	0.899	0.894	0.893	0.899	0.896	0.883	0.913	0.908	0.884	0.892	0.914	0.904	0.907	0.905	0.895	0.905	0.900
	SapBERT	0.882	0.912	0.897	0.896	0.914	0.905	0.916	0.910	0.907	0.921	0.917	**0.919**	0.891	0.906	0.898	0.890	0.899	0.894
		Prior Comparable SOTA (BERT+BiLSTM+CRF with feature engineering, Alamro et al. 2024^7^) = 0.916																	
BC2GM	bert-base-uncased	0.806	0.806	0.806	0.817	0.792	0.804	0.799	0.810	0.805	0.804	0.811	0.808	0.782	0.779	0.781	0.779	0.777	0.778
	distilbert-base-uncased	0.767	0.791	0.779	0.781	0.784	0.783	0.789	0.797	0.793	0.789	0.796	0.793	0.776	0.767	0.772	0.762	0.761	0.762
	roberta-base	0.789	0.787	0.788	0.806	0.813	0.810	0.809	0.806	0.807	0.810	0.813	0.812	0.774	0.778	0.776	0.765	0.766	0.766
	biobert-v1.1	0.837	0.819	0.828	0.828	0.837	0.832	0.836	0.847	0.841	0.837	0.842	0.840	0.807	0.825	0.816	0.792	0.810	0.801
	Bio_ClinicalBERT	0.793	0.793	0.793	0.802	0.801	0.801	0.805	0.815	0.810	0.812	0.819	0.816	0.795	0.802	0.799	0.778	0.791	0.784
	BiomedNLP	0.834	0.824	0.829	0.846	0.834	0.840	0.832	0.848	0.840	0.839	0.845	*0.842*	0.815	0.814	0.814	0.798	0.816	0.807
	SapBERT	0.837	0.827	0.832	0.833	0.843	0.838	0.841	0.842	*0.842*	0.840	0.844	*0.842*	0.814	0.821	0.818	0.798	0.826	0.812
		Prior Comparable SOTA (BioBERT+BiLSTM with embeddings, Alamro et al. 2024^7^) = 0.891																	
JNLPBA	bert-base-uncased	0.786	0.788	0.787	0.770	0.803	0.786	0.777	0.802	0.789	0.779	0.800	0.789	0.782	0.803	0.792	0.774	0.798	0.786
	distilbert-base-uncased	0.766	0.784	0.775	0.782	0.785	0.784	0.750	0.802	0.775	0.750	0.796	0.772	0.779	0.798	0.788	0.775	0.789	0.782
	roberta-base	0.786	0.813	0.800	0.784	0.812	0.797	0.789	0.819	0.804	0.779	0.815	0.797	0.754	0.781	0.767	0.759	0.776	0.768
	biobert-v1.1	0.779	0.818	0.798	0.790	0.806	0.798	0.786	0.812	0.799	0.781	0.822	0.801	0.789	0.816	0.802	0.783	0.814	0.798
	Bio_ClinicalBERT	0.781	0.795	0.788	0.773	0.805	0.788	0.774	0.809	0.791	0.772	0.806	0.789	0.779	0.808	0.793	0.778	0.797	0.787
	BiomedNLP	0.796	0.801	0.799	0.789	0.818	0.804	0.786	0.822	0.804	0.787	0.815	0.801	0.798	0.816	*0.807*	0.787	0.810	0.798
	SapBERT	0.784	0.810	0.797	0.778	0.818	0.798	0.785	0.818	0.801	0.781	0.815	0.797	0.792	0.816	0.804	0.793	0.820	0.806
		Prior Comparable SOTA (BiLSTM+CRF with pretrained word embeddings, Dash et al. 2022^12^) = 0.843																	

DP stands for dependency parsing. DP as Graph corresponds to alternative (1), where the parser output is encoded directly by the RGAT. DP as Seq. Labeling is alternative (2), where it is encoded via sequence labelling, with relative (Rel) and absolute (Abs) encodings. Multi-task Learning corresponds to the joint training of NER and sequence-labelling parsing. Precision (P), Recall (R), and F1-score (F1) are the performance metrics. Bold represents where we beat previous SOTA. Italics is the best F1 result with respect to our experiments

In terms of the models, we observed the best results with SapBERT on most datasets except MTSamples, where bioBERT performed better. Sequence labelling with absolute encoding appears the most beneficial syntax representation to add to the attention mechanism; in the case of VAERS, there is a negligible difference between sequence labelling and the direct graph encoding. We note that VAERS remains one of the most challenging datasets and that with dependency parsing, we were able to improve SOTA by 2.7%. Sequence labelling parsing is meant to combine local and global information about the sentence structure and its elements; our hypothesis corroborated by the results is that it is most beneficial where long distance and local information are comparably important.

We observe the least success with the BC2GM and JNLPBA datasets. These datasets are more homogeneous, but also more technical than MTSamples, VAERS, or NCBI. The named entities they contain are often in the form of number and punctuation sequences. It is possible that the opaqueness/arbitrariness of gene and protein names makes it more difficult to disambiguate between them and other entities, since the role of context with respect to gene names may not be similar to other kinds of entities. Furthermore, we note that the best results achieved on these two datasets include BiLSTM and pretrained word embedding. It makes sense that word embedding would help, since the embedding would offset the negative effect that the opaqueness of gene and protein names may have on the NER task.

## Discussion

**Figure 4.**
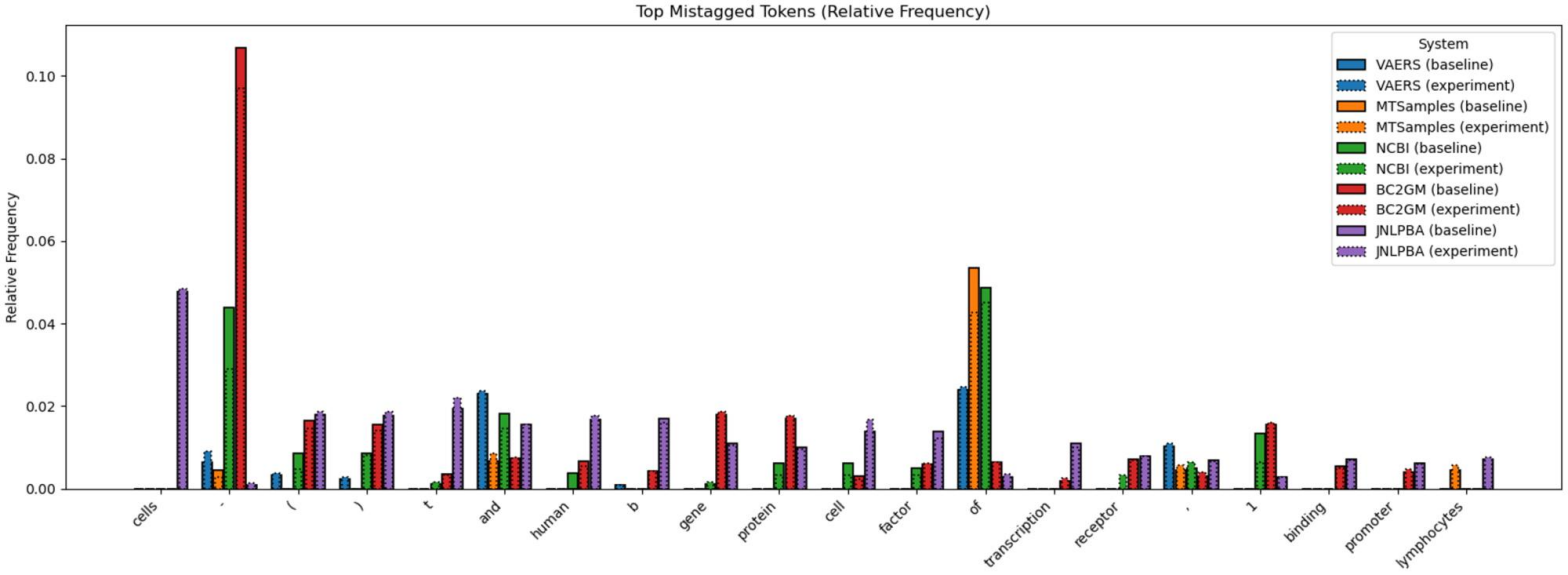
Most mistagged tokens: comparison between our system and the baseline.

**Figure 5.**
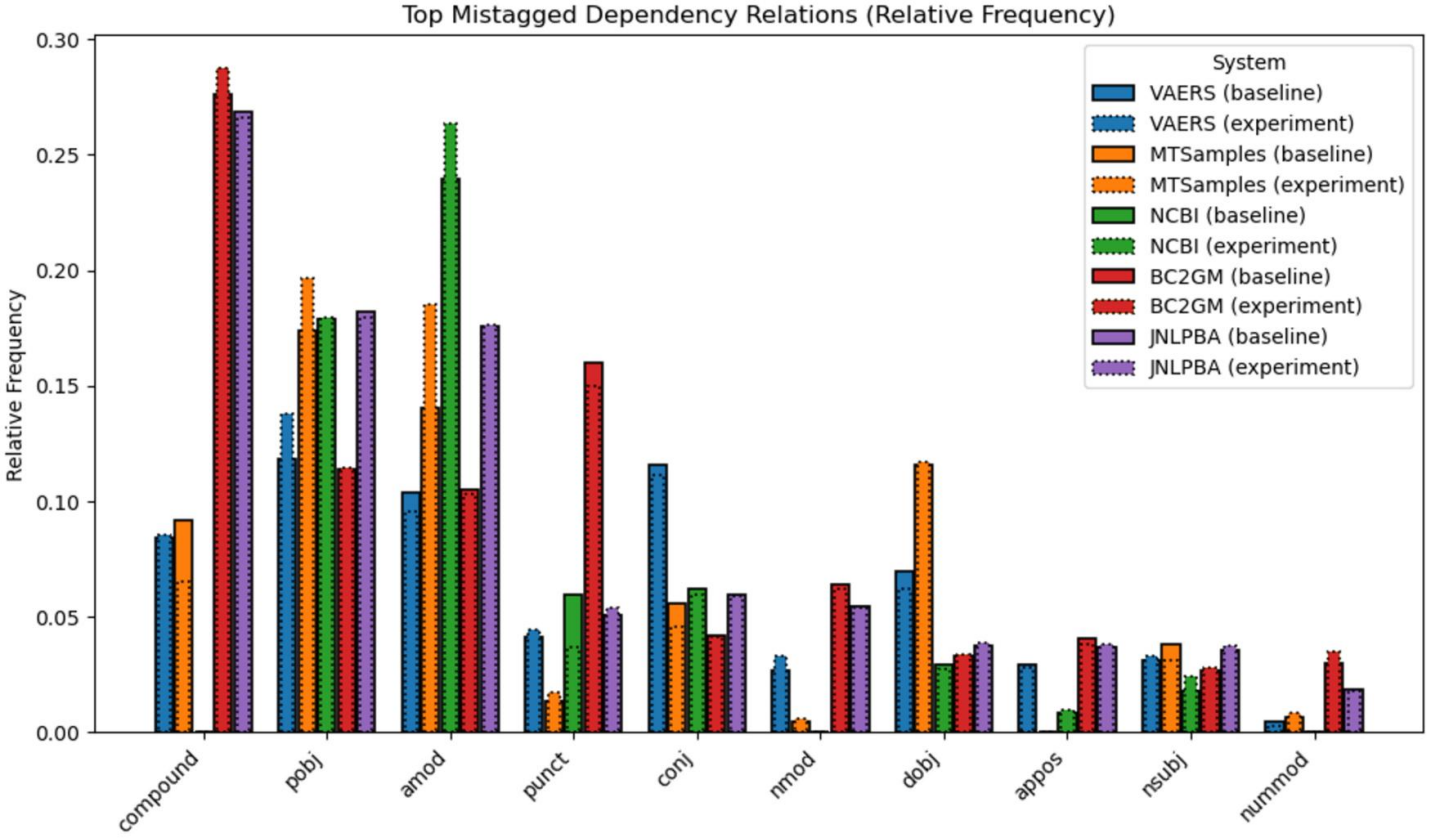
Most mistagged dependency relations: comparison between our system and the baseline.

Table 4 presents the error analysis we performed on the mismatches between the gold labels provided with the datasets and our overall best systems’ predictions, namely dependency parsing as sequence labelling with absolute encodings. We have aggregated the errors based on entity types (in the case of NCBI and BC2GM datasets, there is only a single entity type for each). The example illustrating each category was picked based on the frequency of the mistagged tokens in the given dataset (and secondly, on the sentence length; we picked shorter examples for the table). Pink spans are gold entities; blue spans are predicted entities; finally, underlined spans are those for which the type is different between gold and predicted. Most mistagged tokens per dataset are presented in Figure 4. The only mismatch intersection between all datasets are function words (*and*, *of*) and punctuation. Some of the most noticeable gains are due to now properly tagged n-dash (‘–’) and parentheses, which indicates gains in tagging compounds and appositions, dependency types we do see as most frequently mistagged (Figure 5), although while on some datasets (MTSamples), we get fewer mismatches in these categories compared to the baseline, in other datasets we get more (BC2GM).

**Table 4.** Error analysis

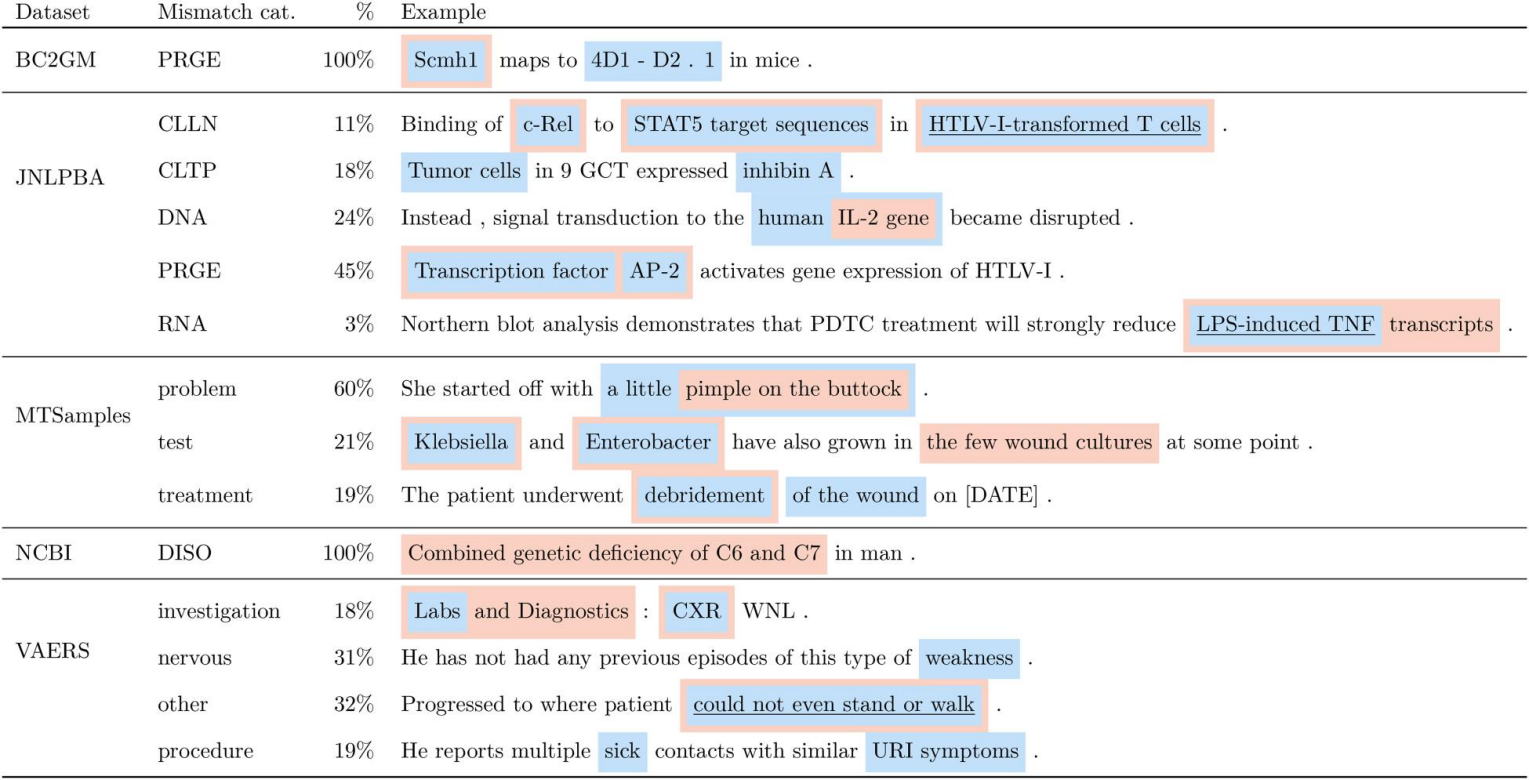

One illustrative example per category. Pink: gold span; blue: predicted span; underlined: mismatched type.

Figure 5 also shows that, while there are few specific mismatch-prone tokens across datasets, there is a similar proportion of mismatches between all datasets with respect to syntactic subjects and ‘prepositional’ objects *nsubj, pobj*, whereas with respect to direct objects (those that do not require a preposition), the MTSamples dataset has noticeably more mismatches than other datasets. It also makes sense that in most datasets (except NCBI) many noun compounds cause mismatches between our system predictions and gold labels, because it is not always clear how to determine what is and what is not a compound; sometimes gold labels interpret as separate entities what our system tags as a single entity, and vice versa. In the end, there is a degree of arbitrariness in this kind of tagging (see also examples of mismatches in Table 4 to assess the degree of arbitrariness).

**Figure 6.**
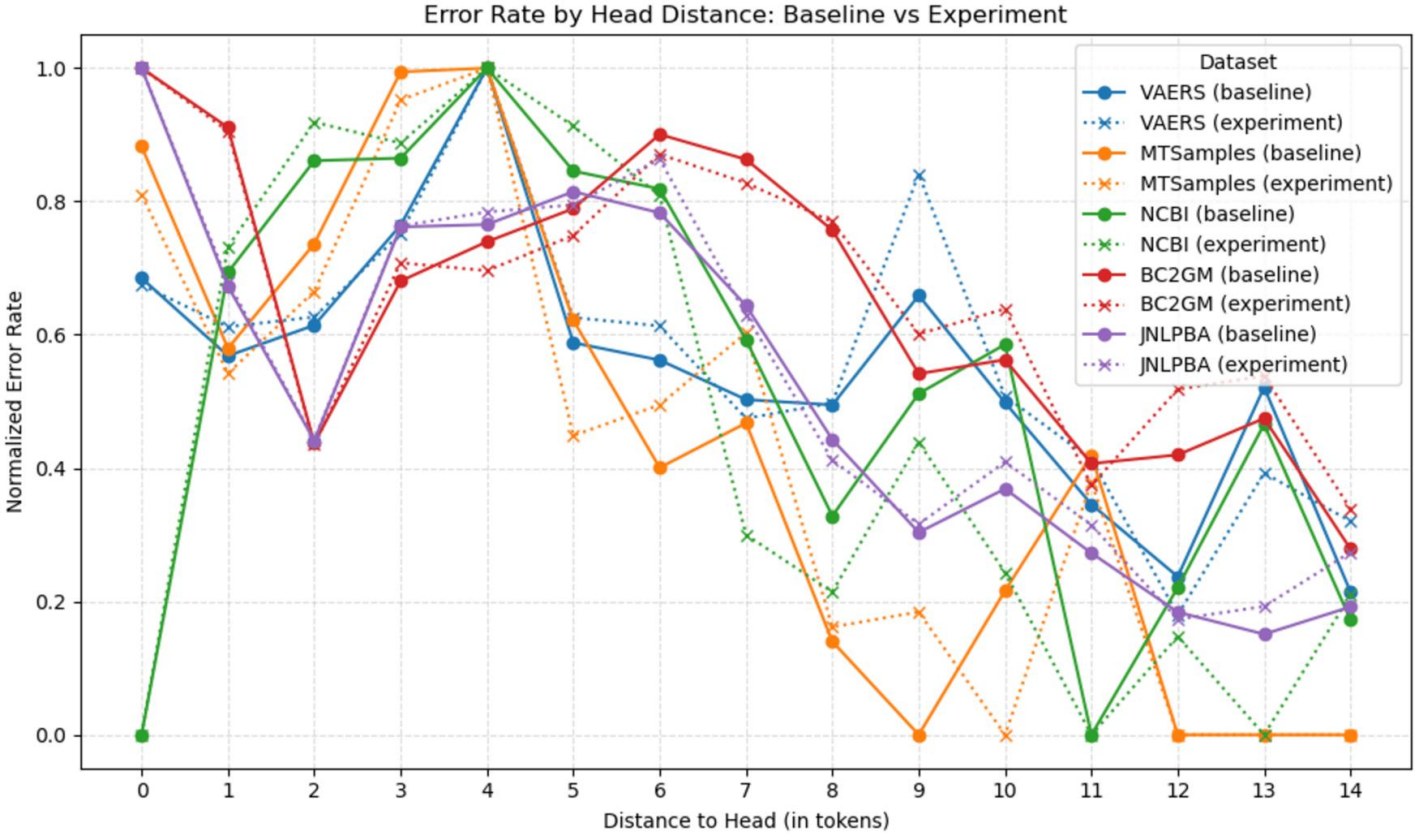
Error rate depending on distance of mistagged token to syntactic head, comparison with baseline.

Figure 6 shows normalized error rates as a function of the mistagged token’s distance to its syntactic head. Intuitively, longer dependencies may indicate higher complexity of utterance, which may correlate with the difficulty of performing NLP tasks on such utterances. This in turn motivates including syntactic features in NLP pipelines. Our experiment shows that including syntactic features does reduce error rates noticeably as distance to head increases in the NCBI dataset, but that we do not see this effect in other datasets. In fact, for BC2GM it seems to be the reverse: syntactic features reduce error rates for shorter distances to the head, but it still increases as the distance grows.

Across categories, function words account for the majority (in some categories, above 50%) of mismatched tokens across datasets. We see many instances of the words *of*, *and*, and *the*, which highlights the strictness of the metric that we apply to evaluation and, related to that, some of the difficulties of obtaining a consistent gold annotation (in the gold annotation, there seems to be a lack of a consistent pattern of including or not including function words in entities). As for substance words in the mismatched entities, there are no specific lexical items that would persist across datasets. This is perhaps not surprising, as it is the diversity and uncommon nature of the named entity array that makes the NER task particularly challenging.

It is worth remarking that false negatives in our system (gold entities that our system misses) are often due to a single error related to the beginning (B) token. For example, in the NCBI example in Table 4, the system successfully identifies each of the tokens in the gold span except the first one, and assigns an I-tag to all of them, while failing to mark the beginning with a B-tag, causing the system to lose the whole entity in evaluation. In other cases, the spans may match perfectly but the first token will not be a B-token but an I-token. This last kind of problem can be solved by simple postprocessing (eg, ensuring that the first tag is always a B-tag), with the potential to further increase accuracy. Both problems may be related to the training data lacking in consistency where it comes to span annotations for similar syntactic structures, ie, sometimes a quantifier (such as ‘some’) is included in the entity and sometimes not, which may confuse the training.

Finally, we note that many of the false positives (where our system tags an entity although the gold annotation marks it as ‘outside’ of any entity span) seem to be cases of questionable gold labelling. Often it is not clear why something would not be tagged in the gold reference. Many of the type mismatches also seem questionable. (This has no bearing on the comparison of our system to others’, as everyone is using the same gold standard.) What this means is that the NER systems such as ours are likely to be better than the current evaluation setups allow us to show. Our error analysis also illustrates some important differences between the datasets: that NCBI is “easy” (various systems will usually score in the 90%s even with strict metrics) because it only has one entity type; that VAERS is “hard” because it has many entity types; and that having many entity types unsurprisingly leads to more inconsistencies in gold label annotations. We hypothesize that higher inconsistency levels of annotation in VAERS could be the reason for the higher percentage of mismatches being due to “wrong type”, compared for example to JNLPBA, which has even more entity types, yet the percentage of mismatches due to wrong type there is lower than in VAERS. Confirming this is future work.

## Conclusion

The models keep improving, and today, a transformer-based model fine-tuned for the NER task outperforms the models which were used in the recent reported SOTA. However, the biomedical datasets remain challenging, with some SOTA still being under 70% F1 score and none approaching 100%. We demonstrate that infusing syntactic information into the attention mechanism of the architecture improves the performance of certain models enough to surpass the previous state of the art on several benchmark datasets, in some cases, up to 5%. The intuition that syntactic structure is important for NER is supported by our error analysis, which shows the large proportion of compounds and adjectival modifiers among errors, as well as function words such as ‘and’ and ‘of’; in other words, complex phrases. Future work should pay attention to the consistency of labelling complex phrases (perhaps employing a syntactic parser as part of the labelling procedure) and explore further opportunities for using linguistic analysis in attention mechanisms of NER systems.

## Supplementary Material

ooaf149_Supplementary_Data

## Data Availability

The code and other resources developed for this work are available in our GitHub repository at: https://github.com/chimran135/SynNER. The datasets used in this study are publicly available from third-party sources. The MTSamples and VAERS datasets can be downloaded from: https://github.com/BIDS-Xu-Lab/Clinical_Entity_Recognition_Using_GPT_models. The NCBI-Disease, BC2GM, and JNLPBA datasets can be downloaded from: https://github.com/cambridgeltl/MTL-Bioinformatics-2016.

## Footnotes

1 Most papers on the topic use entity-based evaluation. Lopez et al. 2025^25^ report 94.75% token-based F1 score on NCBI disease; we get 99% with our sequence labelling setup.

2
https://salsa.grupolys.org/
