# Supplementary material for: SynNER: syntax-infused named entity recognition in the biomedical domain
Source: JAMIA Open. 2026 Feb 21;9(1):ooaf149. doi: 10.1093/jamiaopen/ooaf149 (PMC12932951; doi:10.1093/jamiaopen/ooaf149)

SUPPLEMENTARY MATERIAL

Appendix A

Table A1 presents token-level F1-Score, Table A2 presents boundary-only F1 score for correctly identifying entity spans (irrespective of their entity type), Table A3 presents relax-match entity-level F1-Score and Tables A4–A8 presents the detailed comparison between strict-match and relaxed-match F1-Score at entity-level on all five datasets. Figure A1 and Figure A2 shows tokens and dependency types which account for most error reduction.

Dataset	Encoder	Baseline	DP as Graph	DP as Seq. Labeling		Multi-task Learning	
				Rel	Abs	Rel	Abs
MTSamples	bert-base-uncased	0.9376	0.9416	0.9387	0.9402	0.9337	0.9378
	distilbert-base-uncased	0.9319	0.9362	0.9354	0.9337	0.9372	0.9343
	roberta-base	0.9373	0.9345	*0.9462*	0.9374	0.9328	0.9358
	biobert-v1.1	0.9330	0.9397	0.9403	0.9452	0.9411	0.9431
	Bio_ClinicalBERT	0.9343	0.9340	0.9356	0.9379	0.9369	0.9378
	BiomedNLP	0.9399	0.9378	0.9410	0.9381	0.9467	0.9449
	SapBERT	0.9353	0.9384	0.9388	0.9412	0.9452	0.9399
VAERS	bert-base-uncased	0.8678	0.8782	0.8798	0.8735	0.8723	0.8672
	distilbert-base-uncased	0.8601	0.8643	0.8639	0.8717	0.8690	0.8603
	roberta-base	0.8895	0.8879	0.8894	0.8894	0.8727	0.8758
	biobert-v1.1	0.8804	0.8866	0.8834	0.8860	0.8890	0.8844
	Bio_ClinicalBERT	0.8833	0.8864	0.8896	0.8888	0.8816	0.8793
	BiomedNLP	0.8793	0.8942	0.8826	0.8908	0.8826	0.8836
	SapBERT	0.8878	0.9016	0.8980	*0.9018*	0.8873	0.8830
NCBI	bert-base-uncased	0.9860	0.9869	0.9864	0.9864	0.9866	0.9868
	distilbert-base-uncased	0.9859	0.9857	0.9880	0.9865	0.9857	0.9862
	roberta-base	0.9854	0.9885	*0.9900*	0.9878	0.9865	0.9865
	biobert-v1.1	0.9880	0.9875	0.9882	0.9885	0.9880	0.9879
	Bio_ClinicalBERT	0.9876	0.9875	0.9876	0.9870	0.9872	0.9870
	BiomedNLP	0.9878	0.9885	0.9890	0.9881	0.9887	0.9886
	SapBERT	0.9884	0.9889	0.9856	0.9898	0.9881	0.9876
BC2GM	bert-base-uncased	0.9751	0.9752	0.9753	0.9754	0.9732	0.9717
	distilbert-base-uncased	0.9723	0.9728	0.9739	0.9737	0.9719	0.9696
	roberta-base	0.9736	0.9757	0.9759	0.9748	0.9719	0.9712
	biobert-v1.1	0.9789	0.9801	*0.9806*	0.9804	0.9766	0.9753
	Bio_ClinicalBERT	0.9744	0.9748	0.9765	0.9760	0.9739	0.9725
	BiomedNLP	0.9792	0.9802	0.9799	0.9804	0.9769	0.9752
	SapBERT	0.9789	0.9799	0.9802	0.9803	0.9763	0.9760
JNLPBA	bert-base-uncased	0.9436	0.9451	0.9442	0.9446	0.9453	0.9432
	distilbert-base-uncased	0.9420	0.9446	0.9427	0.9429	0.9434	0.9430
	roberta-base	0.9466	0.9466	0.9464	0.9466	0.9421	0.9424
	biobert-v1.1	0.9479	0.9472	*0.9486*	0.9477	0.9466	0.9465
	Bio_ClinicalBERT	0.9440	0.9455	0.9455	0.9457	0.9447	0.9440
	BiomedNLP	0.9461	0.9485	0.9480	0.9490	0.9481	0.9468
	SapBERT	0.9474	0.9475	0.9477	0.9483	0.9474	0.9477

Table A1. Token-Level Model Performance F1-Scores where rel and abs stands for Relative and Absolute Sequence Labeling Encoding respectively. DP stands for dependency parsing. DP as Graph corresponds to alternative (1), where the parser output is encoded directly by the RGAT. DP as Seq. Labeling is alternative (2), where it is encoded via sequence labeling, with relative (Rel) and absolute (Abs) encodings. Multi-task Learning corresponds to the joint training of NER and sequence-labeling parsing. Italic represents the best results in our experiments.

Table A2. Boundary-Only F1 Scores. DP stands for dependency parsing. DP as Graph corresponds to alternative (1), where the parser output is encoded directly by the RGAT. DP as Seq. Labeling is alternative (2), where it is encoded via sequence labeling, with relative (Rel) and absolute (Abs) encodings. Multi-task Learning corresponds to the joint training of NER and sequence-labeling parsing. Precision (P), Recall (R), and F1-score (F1) are the performance metrics. Italic represents the best result in our experiments.

Dataset	Encoders	Baseline			DP as Graph			DP as Seq. Labeling						Multi-task Learning					
								Rel			Abs			Rel			Abs		
		P	R	F1	P	R	F1	P	R	F1	P	R	F1	P	R	F1	P	R	F1
MTSamples	bert-base-uncased	0.747	0.789	0.767	0.766	0.796	0.781	0.799	0.796	0.797	0.792	0.792	0.792	0.767	0.778	0.773	0.764	0.785	0.774
	distilbert-base-uncased	0.748	0.775	0.761	0.762	0.789	0.775	0.783	0.764	0.774	0.756	0.764	0.760	0.772	0.785	0.778	0.801	0.768	0.784
	roberta-base	0.770	0.768	0.769	0.787	0.768	0.777	0.808	0.813	0.811	0.780	0.810	0.794	0.753	0.771	0.762	0.759	0.764	0.761
	biobert-v1.1	0.742	0.778	0.759	0.801	0.792	0.796	0.795	0.778	0.786	0.819	0.810	*0.814*	0.821	0.806	0.813	0.803	0.789	0.796
	Bio_ClinicalBERT	0.796	0.757	0.776	0.767	0.743	0.755	0.756	0.764	0.760	0.810	0.796	0.803	0.822	0.799	0.811	0.788	0.757	0.772
	BiomedNLP	0.786	0.803	0.794	0.762	0.810	0.785	0.797	0.789	0.793	0.773	0.754	0.763	0.813	0.796	0.804	0.806	0.792	0.799
	SapBERT	0.709	0.771	0.739	0.791	0.771	0.781	0.769	0.796	0.782	0.721	0.775	0.747	0.797	0.817	0.807	0.778	0.789	0.783
VAERS	bert-base-uncased	0.618	0.784	0.691	0.685	0.761	0.721	0.707	0.720	0.714	0.655	0.735	0.693	0.678	0.753	0.713	0.641	0.718	0.677
	distilbert-base-uncased	0.644	0.716	0.678	0.651	0.722	0.685	0.661	0.687	0.674	0.647	0.743	0.692	0.645	0.73	0.685	0.626	0.714	0.667
	roberta-base	0.692	0.804	0.743	0.705	0.807	0.752	0.677	0.788	0.728	0.687	0.790	0.735	0.647	0.763	0.700	0.655	0.741	0.695
	biobert-v1.1	0.662	0.778	0.716	0.695	0.770	0.731	0.671	0.770	0.717	0.691	0.751	0.719	0.719	0.788	0.752	0.696	0.757	0.725
	Bio_ClinicalBERT	0.651	0.747	0.696	0.657	0.768	0.709	0.647	0.761	0.699	0.652	0.763	0.703	0.676	0.737	0.705	0.682	0.747	0.713
	BiomedNLP	0.658	0.753	0.702	0.738	0.805	0.770	0.652	0.798	0.717	0.671	0.782	0.722	0.694	0.770	0.730	0.678	0.761	0.717
	SapBERT	0.700	0.800	0.747	0.721	0.821	0.768	0.707	0.802	0.751	0.735	0.811	*0.772*	0.691	0.788	0.736	0.713	0.784	0.747
NCBI	bert-base-uncased	0.876	0.875	0.876	0.892	0.893	0.893	0.883	0.871	0.877	0.861	0.879	0.870	0.881	0.888	0.885	0.884	0.890	0.887
	distilbert-base-uncased	0.879	0.873	0.876	0.883	0.876	0.879	0.858	0.880	0.869	0.870	0.886	0.878	0.877	0.880	0.879	0.888	0.881	0.884
	roberta-base	0.864	0.894	0.879	0.881	0.909	0.895	0.899	0.899	0.899	0.906	0.896	0.901	0.872	0.882	0.877	0.868	0.877	0.872
	biobert-v1.1	0.893	0.897	0.895	0.894	0.903	0.898	0.894	0.899	0.896	0.900	0.898	0.899	0.891	0.907	0.899	0.894	0.899	0.896
	Bio_ClinicalBERT	0.903	0.881	0.892	0.891	0.892	0.892	0.890	0.901	0.896	0.902	0.876	0.889	0.884	0.883	0.883	0.886	0.875	0.880
	BiomedNLP	0.888	0.899	0.894	0.893	0.899	0.896	0.883	0.913	0.897	0.884	0.892	0.888	0.904	0.907	0.905	0.895	0.905	0.900
	SapBERT	0.882	0.912	0.897	0.896	0.914	0.905	0.916	0.910	0.913	0.921	0.917	*0.919*	0.891	0.906	0.898	0.89	0.899	0.894
BC2GM	bert-base-uncased	0.806	0.806	0.806	0.817	0.792	0.804	0.799	0.810	0.805	0.804	0.811	0.808	0.782	0.779	0.781	0.779	0.777	0.778
	distilbert-base-uncased	0.767	0.791	0.779	0.781	0.784	0.783	0.789	0.797	0.793	0.789	0.796	0.793	0.776	0.767	0.772	0.762	0.761	0.762
	roberta-base	0.789	0.787	0.788	0.806	0.813	0.810	0.809	0.806	0.807	0.810	0.813	0.812	0.774	0.778	0.776	0.765	0.766	0.766
	biobert-v1.1	0.837	0.819	0.828	0.828	0.837	0.832	0.836	0.847	0.841	0.837	0.842	0.840	0.807	0.825	0.816	0.792	0.810	0.801
	Bio_ClinicalBERT	0.793	0.793	0.793	0.802	0.801	0.801	0.805	0.815	0.810	0.812	0.819	0.816	0.795	0.802	0.799	0.778	0.791	0.784
	BiomedNLP	0.834	0.824	0.829	0.846	0.834	0.840	0.832	0.848	0.840	0.839	0.845	*0.842*	0.815	0.814	0.814	0.798	0.816	0.807
	SapBERT	0.837	0.827	0.832	0.833	0.843	0.838	0.841	0.842	*0.842*	0.840	0.844	*0.842*	0.814	0.821	0.818	0.798	0.826	0.812
JNLPBA	bert-base-uncased	0.824	0.825	0.825	0.807	0.842	0.824	0.814	0.840	0.827	0.814	0.837	0.826	0.816	0.839	0.828	0.808	0.834	0.821
	distilbert-base-uncased	0.804	0.822	0.813	0.820	0.823	0.822	0.789	0.844	0.816	0.791	0.840	0.815	0.813	0.833	0.823	0.813	0.828	0.820
	roberta-base	0.820	0.848	0.834	0.817	0.847	0.832	0.820	0.852	0.836	0.815	0.853	0.834	0.788	0.817	0.802	0.795	0.813	0.804
	biobert-v1.1	0.814	0.855	0.834	0.821	0.838	0.829	0.816	0.843	0.830	0.811	0.854	0.832	0.821	0.848	0.834	0.815	0.847	0.831
	Bio_ClinicalBERT	0.818	0.833	0.825	0.809	0.843	0.826	0.812	0.848	0.829	0.810	0.845	0.827	0.815	0.845	0.830	0.813	0.833	0.823
	BiomedNLP	0.827	0.832	0.830	0.822	0.852	0.837	0.818	0.855	0.836	0.817	0.846	0.831	0.831	0.849	*0.840*	0.818	0.842	0.830
	SapBERT	0.817	0.843	0.830	0.813	0.854	0.833	0.818	0.852	0.835	0.813	0.848	0.830	0.826	0.850	0.838	0.822	0.850	0.836

Dataset	Encoder	Baseline	DP as Graph	DP as Seq. Labeling		Multi-task Learning	
				Rel	Abs	Rel	Abs
MTSamples	bert-base-uncased	0.883	0.889	0.896	0.901	0.862	0.886
	distilbert-base-uncased	0.863	0.868	0.865	0.867	0.884	0.876
	roberta-base	0.905	0.875	0.911	0.898	0.876	0.887
	biobert-v1.1	0.881	0.913	0.906	0.91	0.899	0.888
	Bio_ClinicalBERT	0.892	0.889	0.903	0.901	0.873	0.869
	BiomedNLP	0.914	0.910	0.910	0.904	0.915	0.909
	SapBERT	0.878	0.904	0.897	0.895	*0.916*	0.907
VAERS	bert-base-uncased	0.732	0.753	0.736	0.742	0.763	0.734
	distilbert-base-uncased	0.709	0.729	0.705	0.741	0.732	0.714
	roberta-base	0.773	0.787	0.780	0.779	0.746	0.739
	biobert-v1.1	0.780	0.786	0.772	0.791	0.795	0.757
	Bio_ClinicalBERT	0.775	0.784	0.790	0.785	0.765	0.771
	BiomedNLP	0.754	0.788	0.760	0.777	0.774	0.776
	SapBERT	0.773	*0.798*	0.789	*0.798*	0.773	0.780
NCBI	bert-base-uncased	0.940	0.949	0.941	0.942	0.942	0.952
	distilbert-base-uncased	0.944	0.942	0.942	0.946	0.945	0.947
	roberta-base	0.941	0.950	0.953	0.952	0.946	0.944
	biobert-v1.1	0.959	0.954	0.955	0.956	0.955	0.952
	Bio_ClinicalBERT	0.949	0.949	0.955	0.946	0.943	0.940
	BiomedNLP	0.957	0.956	0.960	0.961	0.954	0.954
	SapBERT	0.953	0.958	0.963	*0.964*	0.956	0.957
BC2GM	bert-base-uncased	0.935	0.934	0.941	0.939	0.930	0.925
	distilbert-base-uncased	0.919	0.927	0.930	0.931	0.927	0.918
	roberta-base	0.925	0.942	0.937	0.940	0.929	0.928
	biobert-v1.1	0.952	0.958	0.961	*0.963*	0.949	0.944
	Bio_ClinicalBERT	0.934	0.939	0.943	0.944	0.933	0.927
	BiomedNLP	0.956	0.956	0.960	0.961	0.949	0.944
	SapBERT	0.956	0.960	0.960	0.959	0.951	0.946
JNLPBA	bert-base-uncased	0.877	0.879	0.881	0.879	0.886	0.884
	distilbert-base-uncased	0.871	0.875	0.875	0.874	0.881	0.875
	roberta-base	0.886	0.889	0.890	0.885	0.877	0.877
	biobert-v1.1	0.887	0.891	0.891	0.891	0.891	0.890
	Bio_ClinicalBERT	0.879	0.879	0.879	0.878	0.884	0.880
	BiomedNLP	0.886	0.891	0.893	0.893	0.892	0.891
	SapBERT	0.887	0.888	0.889	0.891	0.891	*0.894*

Table A3. Entity-Level F1-Score (Relaxed Match) where rel and abs stands for Relative and Absolute Sequence Labeling Encoding respectively. DP stands for dependency parsing. DP as Graph corresponds to alternative (1), where the parser output is encoded directly by the RGAT. DP as Seq. Labeling is alternative (2), where it is encoded via sequence labeling, with relative (Rel) and absolute (Abs) encodings. Multi-task Learning corresponds to the joint training of NER and sequence-labeling parsing. Italic represents the best results in our experiments.

Method	Encoding	Encoder	Exact-Match			Relaxed-Match		
			Precision	Recall	F1	Precision	Recall	F1
Baseline	-	bert-base-uncased	0.720	0.761	0.740	0.847	0.923	0.883
		distilbert-base-uncased	0.704	0.729	0.716	0.830	0.898	0.863
		roberta-base	0.749	0.746	0.748	0.898	0.912	0.905
		biobert-v1.1	0.708	0.743	0.725	0.842	0.923	0.881
		Bio_ClinicalBERT	0.774	0.736	0.755	0.907	0.877	0.892
		BiomedNLP	0.769	0.785	*0.777*	0.890	0.940	*0.914*
		SapBERT	0.693	0.754	0.722	0.819	0.947	0.878
DP as Graph	-	bert-base-uncased	0.736	0.764	0.750	0.861	0.919	0.889
		distilbert-base-uncased	0.731	0.757	0.744	0.840	0.898	0.868
		roberta-base	0.773	0.754	0.763	0.877	0.873	0.875
		biobert-v1.1	0.779	0.771	*0.775*	0.904	0.923	*0.913*
		Bio_ClinicalBERT	0.742	0.718	0.730	0.891	0.887	0.889
		BiomedNLP	0.738	0.785	0.761	0.868	0.958	0.910
		SapBERT	0.769	0.750	0.759	0.903	0.905	0.904
Multi-task Learning	rel	bert-base-uncased	0.726	0.736	0.731	0.844	0.880	0.862
		distilbert-base-uncased	0.730	0.743	0.736	0.858	0.912	0.884
		roberta-base	0.722	0.739	0.730	0.849	0.905	0.876
		biobert-v1.1	0.799	0.785	*0.792*	0.903	0.894	0.899
		Bio_ClinicalBERT	0.797	0.775	0.786	0.877	0.870	0.873
		BiomedNLP	0.795	0.778	0.786	0.910	0.919	0.915
		SapBERT	0.780	0.799	0.790	0.893	0.940	*0.916*
	abs	bert-base-uncased	0.736	0.757	0.747	0.856	0.919	0.886
		distilbert-base-uncased	0.765	0.732	0.748	0.886	0.866	0.876
		roberta-base	0.731	0.736	0.733	0.871	0.905	0.887
		biobert-v1.1	0.785	0.771	*0.778*	0.889	0.887	0.888
		Bio_ClinicalBERT	0.755	0.725	0.740	0.864	0.873	0.869
		BiomedNLP	0.781	0.768	0.774	0.903	0.915	*0.909*
		SapBERT	0.760	0.771	0.766	0.889	0.926	0.907
DP as Seq. Labeling	rel	bert-base-uncased	0.767	0.764	0.765	0.883	0.908	0.896
		distilbert-base-uncased	0.747	0.729	0.738	0.874	0.856	0.865
		roberta-base	0.794	0.799	*0.796*	0.906	0.915	*0.911*
		biobert-v1.1	0.773	0.757	0.765	0.899	0.912	0.906
		Bio_ClinicalBERT	0.728	0.736	0.732	0.878	0.930	0.903
		BiomedNLP	0.783	0.775	0.779	0.904	0.915	0.910
		SapBERT	0.745	0.771	0.758	0.867	0.930	0.897
	abs	bert-base-uncased	0.757	0.757	0.757	0.887	0.915	0.901
		distilbert-base-uncased	0.711	0.718	0.715	0.847	0.887	0.867
		roberta-base	0.763	0.792	0.777	0.875	0.923	0.898
		biobert-v1.1	0.804	0.796	*0.800*	0.904	0.915	*0.910*
		Bio_ClinicalBERT	0.785	0.771	0.778	0.900	0.901	0.901
		BiomedNLP	0.758	0.739	0.749	0.906	0.901	0.904
		SapBERT	0.698	0.750	0.723	0.839	0.958	0.895

Table A4. Model Performance on the MTSamples Dataset. DP stands for dependency parsing. DP as Graph corresponds to alternative (1), where the parser output is encoded directly by the RGAT. DP as Seq. Labeling is alternative (2), where it is encoded via sequence labeling, with relative (Rel) and absolute (Abs) encodings. Multi-task Learning corresponds to the joint training of NER and sequence-labeling parsing. Italic represents the best results in our experiments.

Method	Encoding	Encoder	Exact-Match			Relaxed-Match		
			Precision	Recall	F1	Precision	Recall	F1
Baseline	-	bert-base-uncased	0.532	0.675	0.595	0.643	0.850	0.732
		distilbert-base-uncased	0.539	0.599	0.568	0.660	0.765	0.709
		roberta-base	0.610	0.708	*0.655*	0.719	0.837	0.773
		biobert-v1.1	0.588	0.691	0.635	0.707	0.870	*0.780*
		Bio_ClinicalBERT	0.580	0.665	0.620	0.712	0.850	0.775
		BiomedNLP	0.570	0.652	0.608	0.692	0.829	0.754
		SapBERT	0.606	0.693	0.647	0.712	0.846	0.773
DP as Graph	-	bert-base-uncased	0.597	0.663	0.629	0.708	0.805	0.753
		distilbert-base-uncased	0.546	0.605	0.574	0.679	0.788	0.729
		roberta-base	0.625	0.716	0.667	0.732	0.852	0.787
		biobert-v1.1	0.612	0.679	0.644	0.737	0.842	0.786
		Bio_ClinicalBERT	0.582	0.681	0.628	0.715	0.868	0.784
		BiomedNLP	0.649	0.708	0.677	0.750	0.829	0.788
		SapBERT	0.653	0.743	*0.695*	0.744	0.862	*0.798*
Multi-task Learning	rel	bert-base-uncased	0.580	0.644	0.610	0.713	0.821	0.763
		distilbert-base-uncased	0.554	0.626	0.588	0.676	0.798	0.732
		roberta-base	0.568	0.669	0.614	0.682	0.823	0.746
		biobert-v1.1	0.647	0.708	*0.676*	0.751	0.844	*0.795*
		Bio_ClinicalBERT	0.594	0.648	0.620	0.718	0.817	0.765
		BiomedNLP	0.601	0.667	0.632	0.727	0.829	0.774
		SapBERT	0.613	0.698	0.653	0.722	0.833	0.773
	abs	bert-base-uncased	0.549	0.615	0.580	0.684	0.792	0.734
		distilbert-base-uncased	0.520	0.593	0.555	0.659	0.780	0.714
		roberta-base	0.572	0.648	0.608	0.686	0.802	0.739
		biobert-v1.1	0.608	0.661	0.634	0.719	0.800	0.757
		Bio_ClinicalBERT	0.595	0.652	0.622	0.723	0.827	0.771
		BiomedNLP	0.594	0.667	0.629	0.724	0.837	0.776
		SapBERT	0.628	0.691	*0.658*	0.743	0.821	*0.780*
DP as Seq. Labeling	rel	bert-base-uncased	0.595	0.605	0.600	0.719	0.753	0.736
		distilbert-base-uncased	0.545	0.566	0.555	0.678	0.735	0.705
		roberta-base	0.609	0.708	0.655	0.719	0.852	0.780
		biobert-v1.1	0.576	0.661	0.616	0.705	0.854	0.772
		Bio_ClinicalBERT	0.586	0.689	0.633	0.719	0.877	*0.790*
		BiomedNLP	0.568	0.695	0.625	0.676	0.870	0.760
		SapBERT	0.636	0.722	*0.676*	0.732	0.854	0.789
	abs	bert-base-uncased	0.568	0.638	0.601	0.692	0.800	0.742
		distilbert-base-uncased	0.566	0.650	0.605	0.683	0.809	0.741
		roberta-base	0.618	0.710	0.661	0.724	0.842	0.779
		biobert-v1.1	0.608	0.661	0.634	0.748	0.840	0.791
		Bio_ClinicalBERT	0.587	0.687	0.633	0.715	0.870	0.785
		BiomedNLP	0.599	0.698	0.645	0.711	0.856	0.777
		SapBERT	0.661	0.730	*0.694*	0.753	0.848	*0.798*

Table A5. Model Performance on the VARES Dataset. DP stands for dependency parsing. DP as Graph corresponds to alternative (1), where the parser output is encoded directly by the RGAT. DP as Seq. Labeling is alternative (2), where it is encoded via sequence labeling, with relative (Rel) and absolute (Abs) encodings. Multi-task Learning corresponds to the joint training of NER and sequence-labeling parsing. Italic represents the best results in our experiments.

Method	Encoding	Encoder	Exact-Match			Relaxed-Match		
			Precision	Recall	F1	Precision	Recall	F1
Baseline	-	bert-base-uncased	0.876	0.875	0.876	0.937	0.943	0.940
		distilbert-base-uncased	0.879	0.873	0.876	0.945	0.942	0.944
		roberta-base	0.864	0.894	0.879	0.918	0.966	0.941
		biobert-v1.1	0.893	0.897	0.895	0.953	0.965	*0.959*
		Bio_ClinicalBERT	0.903	0.881	0.892	0.960	0.940	0.949
		BiomedNLP	0.888	0.899	0.894	0.945	0.969	0.957
		SapBERT	0.882	0.912	*0.897*	0.932	0.976	0.953
DP as Graph	-	bert-base-uncased	0.892	0.893	0.893	0.947	0.950	0.949
		distilbert-base-uncased	0.883	0.876	0.879	0.943	0.942	0.942
		roberta-base	0.881	0.909	0.895	0.934	0.966	0.950
		biobert-v1.1	0.894	0.903	0.898	0.947	0.961	0.954
		Bio_ClinicalBERT	0.891	0.892	0.892	0.944	0.954	0.949
		BiomedNLP	0.893	0.899	0.896	0.948	0.964	0.956
		SapBERT	0.896	0.914	*0.905*	0.946	0.969	*0.958*
Multi-task Learning	rel	bert-base-uncased	0.881	0.888	0.885	0.935	0.949	0.942
		distilbert-base-uncased	0.877	0.880	0.879	0.939	0.950	0.945
		roberta-base	0.872	0.882	0.877	0.935	0.956	0.946
		biobert-v1.1	0.891	0.907	0.899	0.945	0.966	0.955
		Bio_ClinicalBERT	0.884	0.883	0.883	0.942	0.945	0.943
		BiomedNLP	0.904	0.907	*0.905*	0.950	0.959	0.954
		SapBERT	0.891	0.906	0.898	0.947	0.966	*0.956*
	abs	bert-base-uncased	0.884	0.890	0.887	0.946	0.958	0.952
		distilbert-base-uncased	0.888	0.881	0.884	0.948	0.947	0.947
		roberta-base	0.868	0.877	0.872	0.934	0.954	0.944
		biobert-v1.1	0.894	0.899	0.896	0.947	0.958	0.952
		Bio_ClinicalBERT	0.886	0.875	0.880	0.943	0.937	0.940
		BiomedNLP	0.895	0.905	*0.900*	0.946	0.962	0.954
		SapBERT	0.890	0.899	0.894	0.951	0.964	*0.957*
DP as Seq. Labeling	rel	bert-base-uncased	0.883	0.871	0.877	0.944	0.937	0.941
		distilbert-base-uncased	0.858	0.880	0.869	0.923	0.962	0.942
		roberta-base	0.899	0.899	0.899	0.953	0.953	0.953
		biobert-v1.1	0.894	0.899	0.896	0.951	0.960	0.955
		Bio_ClinicalBERT	0.890	0.901	0.896	0.944	0.966	0.955
		BiomedNLP	0.883	0.913	0.897	0.936	0.985	0.960
		SapBERT	0.916	0.910	*0.913*	0.963	0.962	*0.963*
	abs	bert-base-uncased	0.861	0.879	0.870	0.924	0.961	0.942
		distilbert-base-uncased	0.870	0.886	0.878	0.931	0.962	0.946
		roberta-base	0.906	0.896	0.901	0.957	0.948	0.952
		biobert-v1.1	0.900	0.898	0.899	0.956	0.956	0.956
		Bio_ClinicalBERT	0.902	0.876	0.889	0.957	0.936	0.946
		BiomedNLP	0.884	0.892	0.888	0.951	0.972	0.961
		SapBERT	0.921	0.917	*0.919*	0.965	0.964	*0.964*

Table A6. Model Performance on the NCBI Dataset. DP stands for dependency parsing. DP as Graph corresponds to alternative (1), where the parser output is encoded directly by the RGAT. DP as Seq. Labeling is alternative (2), where it is encoded via sequence labeling, with relative (Rel) and absolute (Abs) encodings. Multi-task Learning corresponds to the joint training of NER and sequence-labeling parsing. Italic represents the best results in our experiments.

Method	Encoding	Encoder	Exact-Match			Relaxed-Match		
			Precision	Recall	F1	Precision	Recall	F1
Baseline	-	bert-base-uncased	0.806	0.806	0.806	0.930	0.939	0.935
		distilbert-base-uncased	0.767	0.791	0.779	0.898	0.940	0.919
		roberta-base	0.789	0.787	0.788	0.923	0.927	0.925
		biobert-v1.1	0.837	0.819	0.828	0.961	0.944	0.952
		Bio_ClinicalBERT	0.793	0.793	0.793	0.927	0.940	0.934
		BiomedNLP	0.834	0.824	0.829	0.960	0.952	*0.956*
		SapBERT	0.837	0.827	*0.832*	0.96	0.952	*0.956*
DP as Graph	-	bert-base-uncased	0.817	0.792	0.804	0.947	0.922	0.934
		distilbert-base-uncased	0.781	0.784	0.783	0.919	0.935	0.927
		roberta-base	0.806	0.813	0.810	0.934	0.951	0.942
		biobert-v1.1	0.828	0.837	0.832	0.948	0.969	0.958
		Bio_ClinicalBERT	0.802	0.801	0.801	0.935	0.942	0.939
		BiomedNLP	0.846	0.834	*0.840*	0.962	0.951	0.956
		SapBERT	0.833	0.843	0.838	0.952	0.969	*0.960*
Multi-task Learning	rel	bert-base-uncased	0.782	0.779	0.781	0.927	0.932	0.93
		distilbert-base-uncased	0.776	0.767	0.772	0.927	0.926	0.927
		roberta-base	0.774	0.778	0.776	0.916	0.942	0.929
		biobert-v1.1	0.807	0.825	0.816	0.936	0.963	0.949
		Bio_ClinicalBERT	0.795	0.802	0.799	0.925	0.941	0.933
		BiomedNLP	0.815	0.814	0.814	0.947	0.951	0.949
		SapBERT	0.814	0.821	*0.818*	0.943	0.959	*0.951*
	abs	bert-base-uncased	0.779	0.777	0.778	0.923	0.928	0.925
		distilbert-base-uncased	0.762	0.761	0.762	0.913	0.923	0.918
		roberta-base	0.765	0.766	0.766	0.920	0.936	0.928
		biobert-v1.1	0.792	0.810	0.801	0.927	0.961	0.944
		Bio_ClinicalBERT	0.778	0.791	0.784	0.918	0.937	0.927
		BiomedNLP	0.798	0.816	0.807	0.931	0.958	0.944
		SapBERT	0.798	0.826	*0.812*	0.926	0.968	*0.946*
DP as Seq. Labeling	rel	bert-base-uncased	0.799	0.810	0.805	0.929	0.953	0.941
		distilbert-base-uncased	0.789	0.797	0.793	0.920	0.94	0.930
		roberta-base	0.809	0.806	0.807	0.937	0.937	0.937
		biobert-v1.1	0.836	0.847	0.841	0.951	0.971	*0.961*
		Bio_ClinicalBERT	0.805	0.815	0.810	0.933	0.954	0.943
		BiomedNLP	0.832	0.848	0.840	0.948	0.973	0.960
		SapBERT	0.841	0.842	*0.842*	0.957	0.962	0.960
	abs	bert-base-uncased	0.804	0.811	0.808	0.933	0.945	0.939
		distilbert-base-uncased	0.789	0.796	0.793	0.922	0.941	0.931
		roberta-base	0.810	0.813	0.812	0.937	0.942	0.940
		biobert-v1.1	0.837	0.842	0.840	0.957	0.969	0.963
		Bio_ClinicalBERT	0.812	0.819	0.816	0.936	0.952	0.944
		BiomedNLP	0.839	0.845	*0.842*	0.957	0.966	*0.961*
		SapBERT	0.840	0.844	*0.842*	0.957	0.962	0.959

Table A7. Model Performance on the BC2GM Dataset. DP stands for dependency parsing. DP as Graph corresponds to alternative (1), where the parser output is encoded directly by the RGAT. DP as Seq. Labeling is alternative (2), where it is encoded via sequence labeling, with relative (Rel) and absolute (Abs) encodings. Multi-task Learning corresponds to the joint training of NER and sequence-labeling parsing. Italic represents the best results in our experiments.

Method	Encoding	Encoder	Exact-Match			Relaxed-Match		
			Precision	Recall	F1	Precision	Recall	F1
Baseline		bert-base-uncased	0.786	0.788	0.787	0.875	0.879	0.877
		distilbert-base-uncased	0.766	0.784	0.775	0.858	0.885	0.871
		roberta-base	0.786	0.813	*0.800*	0.870	0.903	0.886
		biobert-v1.1	0.779	0.818	0.798	0.859	0.917	0.887
		Bio_ClinicalBERT	0.781	0.795	0.788	0.866	0.893	0.879
		BiomedNLP	0.796	0.801	0.799	0.880	0.892	0.886
		SapBERT	0.784	0.810	0.797	0.868	0.907	*0.887*
DP as Graph		bert-base-uncased	0.770	0.803	0.786	0.855	0.905	0.879
		distilbert-base-uncased	0.782	0.785	0.784	0.869	0.881	0.875
		roberta-base	0.784	0.812	0.797	0.869	0.910	0.889
		biobert-v1.1	0.790	0.806	0.798	0.876	0.906	*0.891*
		Bio_ClinicalBERT	0.773	0.805	0.788	0.854	0.905	0.879
		BiomedNLP	0.789	0.818	*0.804*	0.870	0.912	*0.891*
		SapBERT	0.778	0.818	0.798	0.859	0.918	0.888
Multi-task Learning	rel	bert-base-uncased	0.782	0.803	0.792	0.869	0.904	0.886
		distilbert-base-uncased	0.779	0.798	0.788	0.867	0.896	0.881
		roberta-base	0.754	0.781	0.767	0.849	0.907	0.877
		biobert-v1.1	0.789	0.816	0.802	0.873	0.910	0.891
		Bio_ClinicalBERT	0.779	0.808	0.793	0.863	0.906	0.884
		BiomedNLP	0.798	0.816	*0.807*	0.878	0.906	*0.892*
		SapBERT	0.792	0.816	0.804	0.874	0.910	0.891
	abs	bert-base-uncased	0.774	0.798	0.786	0.864	0.905	0.884
		distilbert-base-uncased	0.775	0.789	0.782	0.861	0.889	0.875
		roberta-base	0.759	0.776	0.768	0.856	0.899	0.877
		biobert-v1.1	0.783	0.814	0.798	0.868	0.915	0.890
		Bio_ClinicalBERT	0.778	0.797	0.787	0.865	0.895	0.880
		BiomedNLP	0.787	0.810	0.798	0.874	0.910	0.891
		SapBERT	0.793	0.820	*0.806*	0.875	0.914	*0.894*
DP as Seq. Labeling	rel	bert-base-uncased	0.777	0.802	0.789	0.863	0.900	0.881
		distilbert-base-uncased	0.750	0.802	0.775	0.838	0.916	0.875
		roberta-base	0.789	0.819	*0.804*	0.870	0.910	0.890
		biobert-v1.1	0.786	0.812	0.799	0.869	0.915	0.891
		Bio_ClinicalBERT	0.774	0.809	0.791	0.854	0.905	0.879
		BiomedNLP	0.786	0.822	*0.804*	0.867	0.921	*0.893*
		SapBERT	0.785	0.818	0.801	0.865	0.915	0.889
	abs	bert-base-uncased	0.779	0.800	0.789	0.863	0.897	0.879
		distilbert-base-uncased	0.750	0.796	0.772	0.840	0.911	0.874
		roberta-base	0.779	0.815	0.797	0.860	0.913	0.885
		biobert-v1.1	0.781	0.822	*0.801*	0.861	0.923	0.891
		Bio_ClinicalBERT	0.772	0.806	0.789	0.851	0.905	0.878
		BiomedNLP	0.787	0.815	*0.801*	0.870	0.918	*0.893*
		SapBERT	0.781	0.815	0.797	0.865	0.918	0.891

Table A8. Model Performance on the JNLPBA Dataset. DP stands for dependency parsing. DP as Graph corresponds to alternative (1), where the parser output is encoded directly by the RGAT. DP as Seq. Labeling is alternative (2), where it is encoded via sequence labeling, with relative (Rel) and absolute (Abs) encodings. Multi-task Learning corresponds to the joint training of NER and sequence-labeling parsing. Italic represents the best results in our experiments.

Figure A1. Top token-level gains compared to the baseline (reduction of errors)

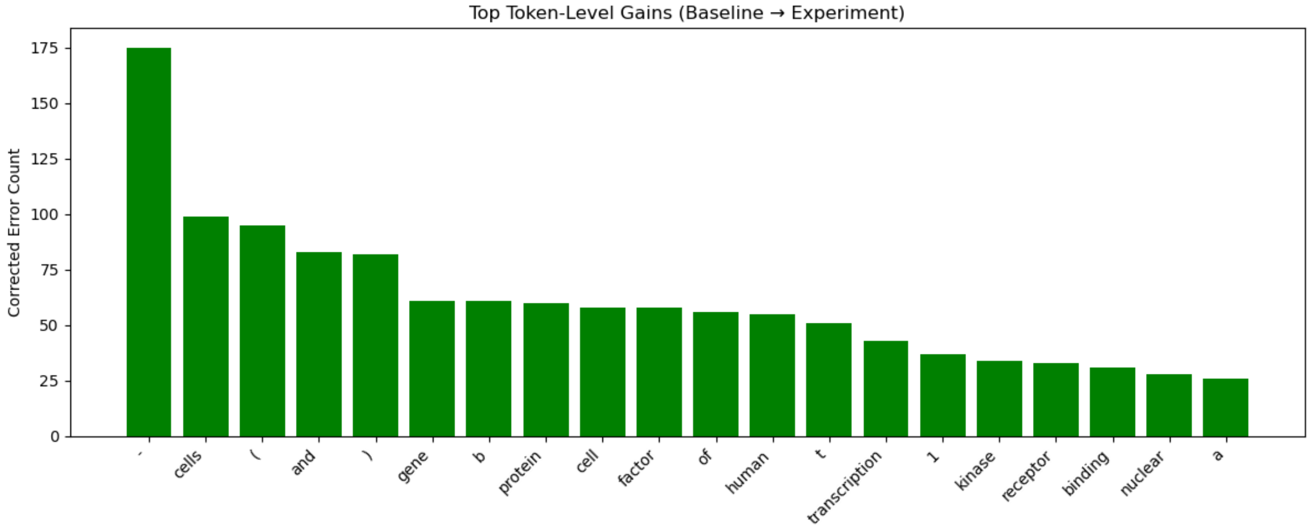

Figure A2. Top dependency-level gains compared to the baseline (reduction of errors)

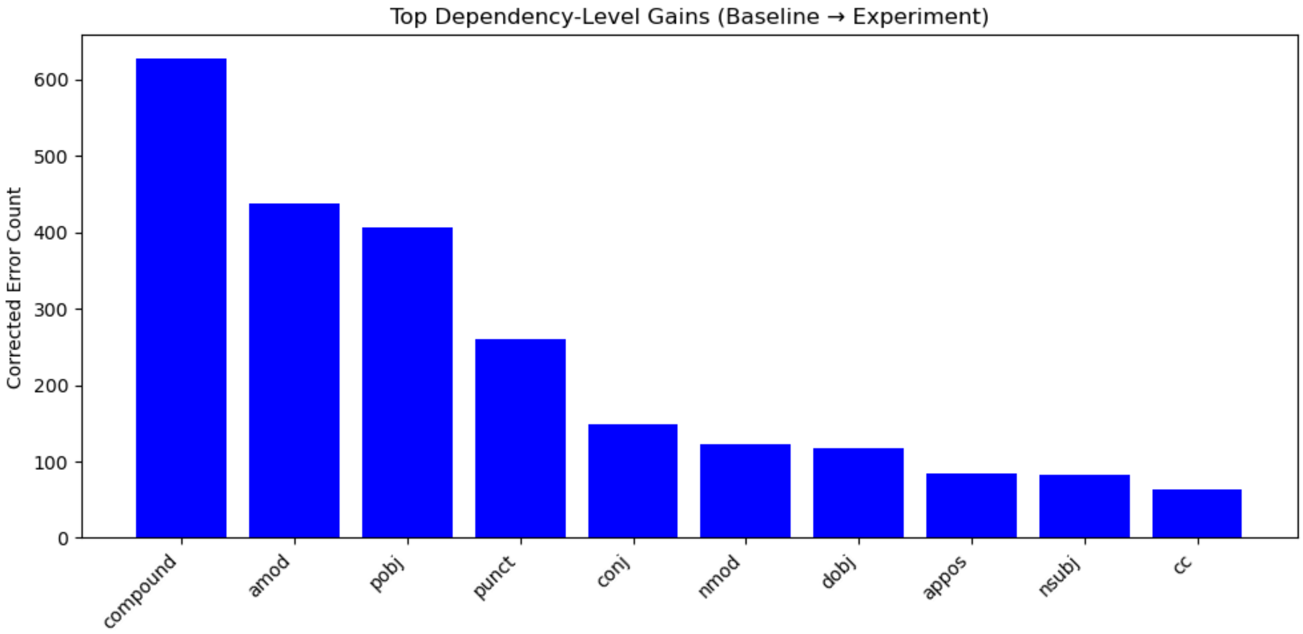

Supplement: ooaf149_Supplementary_Data [file ooaf149_supplementary_data.pdf]
